# iEEG-BIDS, extending the Brain Imaging Data Structure specification to human intracranial electrophysiology

**DOI:** 10.1038/s41597-019-0105-7

**Published:** 2019-06-25

**Authors:** Christopher Holdgraf, Stefan Appelhoff, Stephan Bickel, Kristofer Bouchard, Sasha D’Ambrosio, Olivier David, Orrin Devinsky, Benjamin Dichter, Adeen Flinker, Brett L. Foster, Krzysztof J. Gorgolewski, Iris Groen, David Groppe, Aysegul Gunduz, Liberty Hamilton, Christopher J. Honey, Mainak Jas, Robert Knight, Jean-Philippe Lachaux, Jonathan C. Lau, Christopher Lee-Messer, Brian N. Lundstrom, Kai J. Miller, Jeffrey G. Ojemann, Robert Oostenveld, Natalia Petridou, Gio Piantoni, Andrea Pigorini, Nader Pouratian, Nick F. Ramsey, Arjen Stolk, Nicole C. Swann, François Tadel, Bradley Voytek, Brian A. Wandell, Jonathan Winawer, Kirstie Whitaker, Lyuba Zehl, Dora Hermes

**Affiliations:** 1 The Berkeley Institute for Data Science, Berkeley, USA; 2 Center for Adaptive Rationality, Max Planck Institute for Human Development, Berlin, Germany; 3 Feinstein Institute for Medical Research, Hofstra Northwell School of Medicine, New York, USA; 4 Biological Systems and Engineering Division, Lawrence Berkeley Lab, Berkeley, USA; 5 Università degli Studi di Milano, Milan, Italy; 6 Universite Grenoble Alpes, Inserm, France; 7 NYU School of Medicine, New York, USA; 8 Stanford University, Stanford, USA; 9 Baylor College of Medicine, Houston, Texas USA; 10 New York University, New York, USA; 11 Krembil Research Institute, Toronto, USA; 12 University of Florida, Gainesville, USA; 13 The University of Texas at Austin, Austin, USA; 14 Johns Hopkins University, Baltimore, USA; 15 Martinos Center for Biomedical Imaging, Massachusetts General Hospital, Harvard Med. School, Charlestown, USA; 16 University of California at Berkeley, Berkeley, USA; 17 Centre de Recherche en Neurosciences de Lyon, INSERM, Lyon, France; 18 Department of Clinical Neurological Sciences, Division of Neurosurgery, Western University, London, Canada; 19 Mayo Clinic, Rochester, USA; 20 Department of Neurosurgery, Mayo Clinic, Rochester, USA; 21 University of Washington, Washington, USA; 22 Radboud University, Donders Institute for Brain, Cognition and Behaviour, Karolinska Institutet, Nijmegen, The Netherlands; 23 Center for Image Sciences, University Medical Center Utrecht, Utrecht, The Netherlands; 24 UMC Utrecht Brain Center, Utrecht, The Netherlands; 25 David Geffen School of Medicine at UCLA, California, USA; 26 University of Oregon, Oregon, USA; 27 CHU Grenoble Alpes, GIN, Grenoble, France; 28 UC San Diego, San Diego, USA; 29 Alan Turing Institute, London, UK; 30 Institute for Neuroscience and Medicine (INM-1), Forschungszentrum Jülich GmbH, Jülich, Germany; 31 Department of Physiology & Biomedical Engineering, Mayo Clinic, Rochester, USA; 32 Department of Psychiatry, University of Cambridge, Cambridge, UK

**Keywords:** Software, Cognitive neuroscience, Data publication and archiving

## Abstract

The Brain Imaging Data Structure (BIDS) is a community-driven specification for organizing neuroscience data and metadata with the aim to make datasets more transparent, reusable, and reproducible. Intracranial electroencephalography (iEEG) data offer a unique combination of high spatial and temporal resolution measurements of the living human brain. To improve internal (re)use and external sharing of these unique data, we present a specification for storing and sharing iEEG data: iEEG-BIDS.

Human intracranial electroencephalography^1,2^ (iEEG) data are recorded at specialized medical centers with electrodes placed on or implanted in the human brain^3,4^. Electrodes can be placed during epilepsy monitoring, tumor surgery, or for deep brain stimulation (DBS). During these times, patients can voluntarily participate in scientific experiments^5–9^. Due to the specialized clinical setting in which iEEG data are recorded, iEEG is relatively rare compared to EEG or MRI, and researchers sometimes have to wait several years before completing a study. This makes it important to have a consistent way of archiving and documenting iEEG data within a lab. In addition, because of its unique spatiotemporal properties^5–9^ and the high cost of acquisition, it is important that these unique data, contributed by rare patients, are made maximally useful and reusable to the scientific community.

Currently, iEEG datasets are stored in many different structures, with each lab adopting their own methods for data and metadata organization, which imposes a substantial barrier to collaboration, reproducibility, and scientific progress. In addition, there are many electrophysiology file formats, which do not generally include all information needed to understand, analyze, and reproduce scientific results. This includes the electrode position, referencing method prior to digitization, simultaneously recorded physiology data, the timing of task events, details on the presented stimuli, or anatomical imaging data from the same subjects. There is a clear need for a community standard to better describe all aspects of iEEG data and its experimental context.

BIDS comprises a standardized specification for folder and file naming, the choice of data formats, and the representation of metadata. It is a modality-agnostic specification, relying on community-driven processes to extend the original specification (written for MRI) to new modalities. For example, the MEG community has extended BIDS for this type of data^2^, and the EEG community has recently finished preparing their extension^10^.

This paper announces the extension of the BIDS specification to raw, unprocessed human iEEG data, spanning stereo EEG (sEEG), electrocorticography (ECoG), and deep brain stimulation (DBS). It describes the global BIDS structure and some highlights of the current iEEG-BIDS specification. Adoption of iEEG-BIDS will minimize the burden of data curation, facilitate multimodal data integration, and make iEEG data more valuable to future researchers working in the specific lab and to the wider scientific community.

## iEEG-BIDS Data Structure

**Fig. 1:**
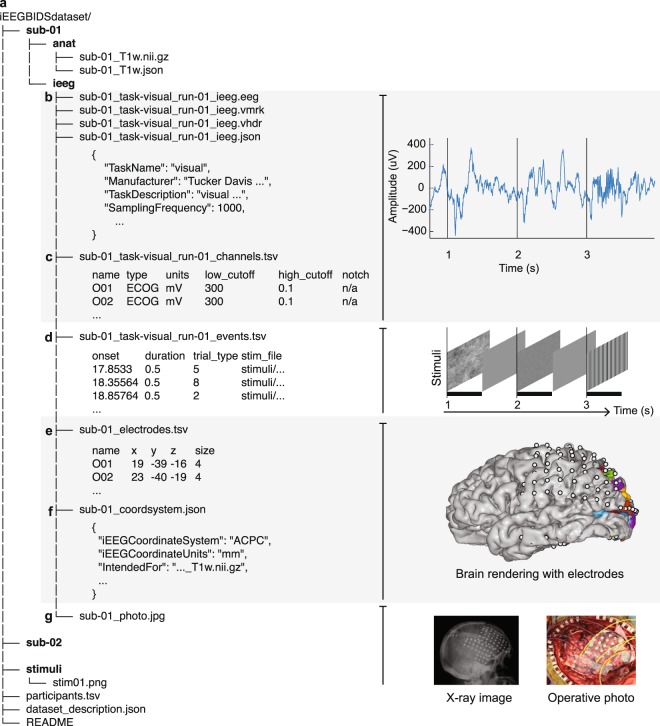
iEEG-BIDS folder structure with pictures of data types. (**a**) This BIDS structure contains several folders for subjects and one for stimuli. Within a subject folder, an /anat/ folder may contain an MRI of a subject alongside iEEG data. Extending the BIDS specification to iEEG data involved the definition of the files in the /ieeg/ folder including new file types and pieces of metadata. (**b)** iEEG data are stored in the BrainVision Core Data format alongside information for acquisition systems and their parameters in an _ieeg.json file. (**c)** Metadata about channel-specific information, such as hardware filters or electrophysiological units are stored in a _channels.tsv file. (**d)** Events during an iEEG recording. Event timing data is stored in an _events.tsv TSV file (**e)** Electrode coordinates are stored in an _electrodes.tsv files and the coordinate system is stored in a _coordsystem.json file. If electrode coordinates are in 3D and are intended for a specific anatomical volume images (.nii.gz), this allows automatically making surface renderings with electrodes and displaying labels from different atlases, here shows with probabilistic maps of visual areas^17^. (**f)** Other images that are relevant for iEEG, such as surface models and 2-D images can be stored in a systematic manner. Optional folders and labels, such as the session folder and space- label, are mostly left out of this example. For other examples, see the BIDS examples: github.com/bids-standard/bids-examples.

The general BIDS specification is designed to modularize data such that it can gracefully handle multiple modalities and recording devices that belong to a single dataset. Metadata are stored either as a Tab Separated Value (TSV) file for tabular data (.tsv) or as JavaScript Object Notation (JSON) file for “key-value” data (.json) (Fig. 1). These file formats have the advantage of being both human and machine-readable. Metadata fields that are not specific to the iEEG extension are shared with the broader BIDS specification. There were several new types of metadata files created for iEEG-BIDS, as well as additions to pre-existing metadata standards in BIDS. These include developing the file content and naming of the metadata for the iEEG data files (_ieeg.json, Fig. 1b), amplifier settings for each channel (_channels.tsv, Fig. 1c), as well as the metallic electrode contacts (_electrodes.tsv) and their coordinate space with a link to a specific reference image (_coordsystem.json) (Fig. 1e). There were several discussed topics that are of particular relevance to the iEEG community, which we briefly cover next.

## Channels vs. Electrodes

Terms for *electrodes* (a physical object), and *channels* (a part of an acquisition system) can potentially overlap, and several definitions were discussed and added as a part of the iEEG-BIDS and EEG-BIDS specifications. Input from members of these communities (as well as those from others such as MEG) resulted in the following definitions for “electrode” and “channel”:

- *Channel: a single analog-digital converter in the recording system that regularly samples the value of a transducer, which results in a signal being represented as a time series in the data. This can be connected to two electrodes (to measure the potential difference between them), a magnetic field or magnetic gradient sensor, temperature sensor, accelerometer, etc*.
- *Electrode: a single point of contact between the acquisition system and the recording site (e.g., scalp, neural tissue,…). Multiple electrodes can be organized as caps (for EEG), arrays, grids, leads, strips, probes, shafts, etc*.

## Electrophysiology Units

As the original BIDS specification dealt primarily with data from an MRI machine, it was also necessary to create standards around the units to be used for electrophysiology data. This was done in partnership with the EEG-BIDS and MEG-BIDS communities. Physical units of any data in a BIDS structure are presented according to their SI unit symbol and possibly prefix symbol (e.g., mV, µV, with the µ as unicode U + 00B5), now specified in the BIDS specification (https://bids-specification.readthedocs.io/en/latest/99-appendices/05-units.html).

## Electrode Locations in iEEG-BIDS

A necessary component to interpret iEEG data are the electrode locations on the cortical surface or embedded in brain tissue. Electrode locations are generally obtained by integrating data from a CT, post-operative MRI, X-ray, or operative photo. Electrodes are then identified and sorted according to medical records, such as a sketch made by the neurological or neurosurgical team. There are several semi-automated software packages that allow researchers to do this. The iEEG-BIDS specification requires that any electrode locations are paired with explicit coordinate systems and, where applicable, the path to an image that can be used to visualize the electrodes on a brain. These may be volume files (e.g., .nii.gz), cortical surfaces, or 2-D images of the patient’s brain. To accommodate localization of iEEG electrodes, iEEG-BIDS added several new options for localization metadata in “_electrodes.tsv” and “_coordsystem.json” files. Electrodes can be localized in multiple spaces by specifying multiple JSON files, and the IntendedFor field makes it straightforward to pair electrode position data with its corresponding image. Finally, iEEG-BIDS explicitly allows for electrode positions to be given in 2-dimensional space in the event that anatomical data are only provided in the form of either operative photos or cortical surface photos.

## Data Formats for iEEG

A challenge in extending the BIDS format to iEEG data was handling the diversity of data formats. Contrary to the MRI community, there is no single accepted format used across iEEG groups. In choosing the data formats considered to be compliant with iEEG-BIDS, the community attempted to cover as many possible iEEG data use-cases with as few unique formats as possible, and an informal survey was sent to the electrophysiology communities to help determine which formats were most suitable as “first class citizens” in the iEEG-BIDS specification. A description of this process and the results was originally published in a blog post for the Berkeley Institute for Data Science (https://bids.berkeley.edu/news/bids-megeegieeg-data-format-survey).

After reviewing community feedback and discussing with the MEG-BIDS and EEG communities^10^, the iEEG-BIDS community decided to support two classes of data formats: compliant formats, and unofficially-supported formats (see the blog post).

### Compliant formats

There are two compliant data formats for the iEEG-BIDS extensions: European Data Format (https://en.wikipedia.org/wiki/European_Data_Format, .edf files, and BrainVision Core Data Format (.vhdr, .vmrk, and eeg) (https://www.brainproducts.com/productdetails.php?id=21&tab=5). They have been chosen because they jointly cover the majority of use-cases in iEEG while also providing a solution for most major technical challenges in iEEG data. EDF is a prolific format in the iEEG community and offers variable sampling rates per channel. BrainVision is supported by EEG-BIDS, and makes up for some technical shortcomings of EDF (such as being able to store data to a higher level of numerical precision of up to 32 bits). Compliant formats in iEEG-BIDS will continue to be supported in the future, and will have the majority of community effort and development in creating tooling.

### Unofficially supported formats

There are three “unofficially-supported” formats. Their use is discouraged, though allowed for reasons stated below. These unofficially-supported formats are: Neurodata Without Borders (.nwb) due to its shared goals in facilitating sharable and reproducible neurophysiology datasets, EEGLab (.set) due to its prevalent use in the iEEG community, and Multiscale Electrophysiology Format version 3 (MEF3) due to its suitability and future potential for clinical iEEG recordings and device-related applications (such as HIPAA-compliant multi-layer encryption of sensitive data). Unofficially-supported formats will pass the validator, but are not guaranteed to become supported in the future, and unofficial support may be dropped.

There is no hard rule about the number of compliant data formats allowed, though the number will be kept as low as possible. Unofficially supported formats may be dropped (if they offer redundant functionality or if their usage drops significantly), or promoted to compliant format status (if they fill a specific niche and meet the guidelines for compliant format status). This process will be carried out by the iEEG-BIDS community in future extension proposals.

## Community Software, Datasets, and Adoption

BIDS is a community project with two primary products: a specification for organizing neuroscience data, and a collection of software and tooling that facilitates the use of BIDS data structures. Creating the iEEG-BIDS specification involved the development of several new community tools. Below we list those that required the most new development for iEEG-BIDS.

### The BIDS validator

A validator (written in javascript) determines whether a given dataset adheres to the BIDS specification (bids-standard.github.io/bids-validator/). It parses a folder, checks whether its hierarchy and naming structure conform to BIDS, and checks text files for the proper metadata and file types needed for the BIDS specification. The BIDS validator was modified in order to validate the new metadata fields and folder structures introduced by the iEEG-BIDS extension.

### Online iEEG datasets organized according to iEEG-BIDS

As a part of creating the iEEG-BIDS specification, five datasets were converted to BIDS format and added to the BIDS-examples github repository (github.com/bids-standard/bids-examples). These examples are meant to demonstrate how the BIDS specification maps onto different scientific use-cases. The neural data of each dataset is removed in order to make it easier to download, though instructions are provided for accessing the full datasets. The datasets available include: an auditory filtered speech experiment, a motor-movement dataset, a visual stimulus dataset, seizure sEEG recordings from a focal epilepsy of the left temporo-occipital junction, and a multimodal dataset with fMRI and ECoG per subject.

### The BIDS starter kit

The BIDS starter kit is a collection of community-driven guides, tutorials, helper scripts, and wiki resources to help researchers get started with the BIDS data structure (github.com/bids-standard/bids-starter-kit). These resources cover the two main languages supported by iEEG-BIDS (Matlab and Python). A tutorial that describes how to create a BIDS-compatible iEEG dataset has been provided on the starter-kit wiki (http://github.com/bids-standard/bids-starter-kit/wiki/Creating-a-BIDS-compatible-iEEG-dataset) and Matlab and Python scripts are also available to produce iEEG sidecar .json and .tsv files.

### pybv and pyedf

While many neuroscience toolboxes exist for the Matlab language, there are relatively fewer for Python. To improve Python support for the primary data formats supported by iEEG-BIDS, two new python packages were created for writing BrainVision (pybv, github.com/bids-standard/pybv) and European Data Format (github.com/bids-standard/pyedf) files. These will be maintained by the BIDS community.

### MNE-BIDS and MNE-Python

The MNE-Python package is an open-source tool for electrophysiology analysis, visualization, and data representation in Python, and has recently been extended to include functionality for iEEG. Alongside the creation of the iEEG-BIDS specification, the community worked alongside those in the EEG and MEG communities to create a new package for moving between MNE-Python and the BIDS specifications in electrophysiology: MNE-BIDS (github.com/mne-tools/mne-bids). MNE-BIDS facilitates converting electrophysiology workflows in MNE-Python into the BIDS dataset framework^11^.

### FieldTrip

FieldTrip is an open toolbox for electrophysiology analytics in the Matlab language^12,13^. It supports importing data from a large number of iEEG formats and can export data to BIDS recommended formats. Furthermore, FieldTrip includes the *data2bids.m* function to help users to organize their iEEG and MRI data and to provide proper metadata annotation (www.fieldtriptoolbox.org/example/bids).

### iElvis

iELVis (github.com/iELVis/iELVis) is an open source Matlab package for localizing iEEG electrodes and visualizing their data overlaid on neuroimaging^14^. It can read and write iEEG electrode coordinates and associated neuroimaging files using BIDS-iEEG conventions. See the BIDS-iEEG compatibility page for more information (http://ielvis.pbworks.com/w/page/130759893/BIDS-iEEG%20Compatibility).

### Brainstorm

Brainstorm is an open-source Matlab application for the analysis of multimodal neurophysiology data^15^. It provides user interfaces for positioning electrode contacts in post-implantation CT or T1 scans as well as reviewing/annotating long data records such as clinical iEEG signals (neuroimage.usc.edu/brainstorm/Tutorials/Epileptogenicity). It supports most file formats commonly used in iEEG and can convert them into BrainVision or EDF files. Studies in Brainstorm’s database can be imported and exported automatically as BIDS-formatted datasets.

### OpenNeuro

Open Neuro is a repository for public neuroimaging data, currently supporting MRI and MEG (OpenNeuro.org). It heavily capitalizes on the BIDS standard - each dataset is validated prior to upload using the bids-validator. OpenNeuro now supports, validates, and accepts iEEG-BIDS data. This allows researchers that adopted BIDS in their labs to easily archive and share their iEEG datasets online.

## BIDS Extensions

The BIDS specification is an ongoing process with a growing community. It uses the BIDS Extension Proposals (BEP) process to discuss and integrate improvements in the BIDS specification. Several outstanding questions will require further community input and discussion, such as formally describing coordinate systems, merging metadata across modalities, and handling derivatives of raw iEEG data. In particular, further discussion is warranted to adapt iEEG-BIDS for non-human subjects and clinical iEEG recordings. As projects such as BIDS make datasets easier to discover and use, it is particularly important to protect the privacy of subjects. BIDS does not directly address privacy concerns - it only provides a structure for how the data should be stored. It is possible for data to be stored with BIDS in a manner that does not divulge subject identity (for example, by removing identifiable images and stripping any patient information from the data file), but this is up to the researcher. Future extensions to the BIDS specification, and documentation from the BIDS community, could help guide researchers in ensuring the privacy of the individuals that have provided their data in a BIDS dataset.

This process will be further improved as the BIDS community continues to grow. Data sharing, reproducibility, and quality control are priority areas of large research consortia, such as the US Brain Research through Advancing Innovative Neurotechnologies (BRAIN) initiative as well as the Human Brain Project (HBP). Within these efforts it has become clear that community-wide accepted data standards are essential^16^. Within the HBP, iEEG-BIDS has been adopted in the Medical Informatics Platform (where it is intended to be deployed in several hospitals for conducting multicenter studies) (http://humanbrainproject.eu/en/medicine/) as well as the Neuroinformatics Platform (where it is supported in the HBP data sharing platform as well as the HBP interactive atlas viewers) (https://www.humanbrainproject.eu/en/explore-the-brain/neuroinformatics-platform/). These institutional stakeholders will be critical in spreading the adoption of BIDS, and encouraging more voices to guide the evolution of the specification moving forward.

## Conclusion

iEEG-BIDS specifies a structured way of storing iEEG data and metadata. BIDS is a community-driven project, and the iEEG-BIDS specification was created after many months of open discussion with the broader iEEG community. One of the core benefits of adopting community standards such as iEEG-BIDS is that it provides a common point that can be the basis for future collaborations. It facilitates reproducibility and cross-modal integration across datasets, experiments, and recording sites.
